# Microwave spectra, molecular geometries, and internal rotation of CH_3_ in *N*-methylimidazole⋯H_2_O and 2-methylimidazole⋯H_2_O Complexes†

**DOI:** 10.1039/d1cp05526g

**Published:** 2022-05-06

**Authors:** Eva Gougoula, Charlotte N. Cummings, Chris Medcraft, Juliane Heitkämper, Nicholas R. Walker

**Affiliations:** Deutsches Elektronen-Synchrotron DESY Notkestr. 85 22607 Hamburg Germany; Chemistry-School of Natural and Environmental Sciences, Newcastle University Bedson Building Newcastle-upon-Tyne NE1 7RU UK nick.walker@newcastle.ac.uk; School of Chemistry, UNSW Sydney Sydney New South Wales 2052 Australia; Institute of Theoretical Chemistry, University of Stuttgart Pfaffenwaldring 55 D-70569 Stuttgart Germany

## Abstract

Broadband microwave spectra have been recorded between 7.0 and 18.5 GHz for *N*-methylimidazole⋯H_2_O and 2-methylimidazole⋯H_2_O complexes. Each complex was generated by co-expansion of low concentrations of methylimidazole and H_2_O in argon buffer gas. The rotational spectra of five isotopologues of each complex have been assigned and analysed to determine rotational constants (*A*_0_, *B*_0_, *C*_0_), centrifugal distortion constants (*D*_*J*_, *D*_*JK*_) and parameters that describe the internal rotation of the CH_3_ group. The results allow the determination of parameters in the (*r*_0_) molecular geometry of each complex. H_2_O is the hydrogen bond donor and the pyridinic nitrogen of imidazole is the hydrogen bond acceptor in each case. The ∠(O–H_b_⋯N3) angles are 177(5)° and 166.3(28)° for *N*-methylimidazole⋯H_2_O and 2-methylimidazole⋯H_2_O respectively. These results are consistent with the presence of a weak electrostatic interaction between the oxygen atom of H_2_O and the hydrogen atom (or CH_3_ group) attached to the C2 carbon atom of imidazole, and with the results of density functional theory calculations. The (*V*_3_) barrier to internal rotation of the CH_3_ group within *N*-methylimidazole⋯H_2_O is essentially unchanged from the value of this parameter for the *N*-methylimidazole monomer. The same parameter is significantly higher for the 2-methylimidazole⋯H_2_O complex than for the 2-methylimidazole monomer as a consequence of the weak electrostatic interaction between the O atom and the CH_3_ group of 2-methylimidazole.

## Abstract 2

Broadband microwave spectra have been recorded between 7.0 and 18.5 GHz for *N*-methylimidazole⋯H_2_O and 2-methylimidazole⋯H_2_O complexes.

## Graphical abstract

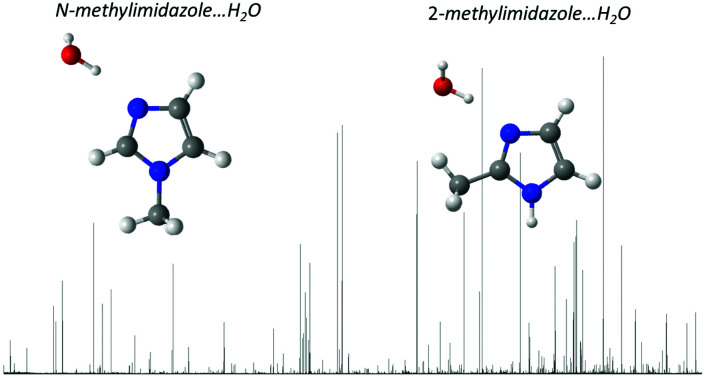

## 1. Introduction

Imidazole rings are found within the anti-fungal drug clotrimazole,^1^ the antibiotic and antiprotozoal drug metronidazole^2^ and various antivirals inhibiting replication of hepatitis viruses (HAV, HBV, HCV *etc*.).^3^ In biological environments, these molecules primarily interact with others through hydrogen bonds. Gas phase spectroscopic experiments provide microscopic and selective insight into individual hydrogen bonding interactions. It is thereby possible to distinguish the microscopic properties of individual hydrogen bonds from properties that result from synergistic (cooperative or competitive) effects that operate at the level of an entire network.

**Fig. 1:**
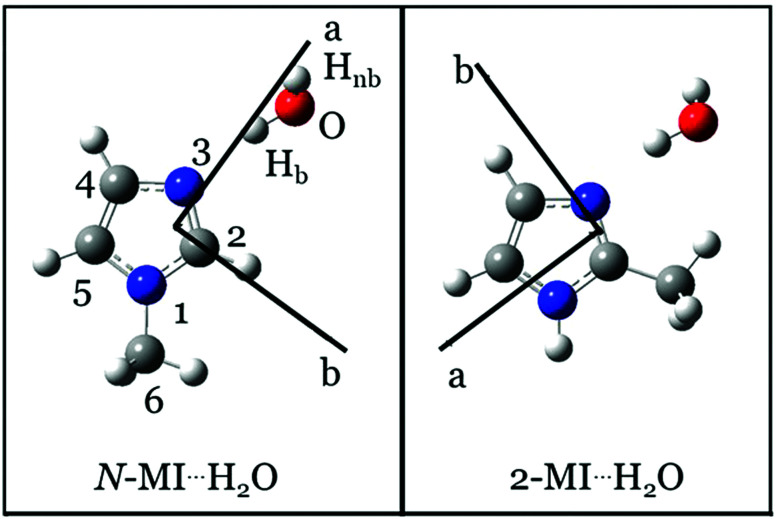
Equilibrium (*r*_e_) geometries of *N*-MI⋯H_2_O (left) and 2-MI⋯H_2_O (right) calculated at the ωB97XD/aug-cc-pVQZ level.

A recent work by our group described the geometries of two isomers of a complex formed between imidazole and H_2_O.^4^ One of these isomers (denoted as imid⋯H_2_O) contains a hydrogen bond between H_2_O and the pyridinic nitrogen, N3, of the imidazole ring (atom numbering of imidazole is shown in Fig. 1). This work and recent results from theoretical calculations^5^ confirm similarities between the structure of imid⋯H_2_O and those of other complexes formed of water and a heteroaromatic molecule such as thiazole,^6^ isoxazole^7^ or pyrimidine.^8^ Specifically, structures of these complexes usually feature H_2_O acting as a proton donor, a heteroatom of the ring acting as a proton acceptor and a secondary interaction where the oxygen atom of water binds to a hydrogen atom of a C–H group on the ring. The present work will show that the geometry of a complex formed of 2-methylimidazole (2-MI) and water is determined by interactions which are very similar to those described above. However, in this case, the secondary interaction is between the oxygen atom of H_2_O and the CH_3_ group attached to C2 of the imidazole ring.

An aim of the present work is to perform the first experimental determination of the change in a (*V*_3_) barrier to internal rotation of CH_3_ which is caused by formation of an electrostatic interaction between H_2_O and a CH_3_ group. It thus begins experimental investigation of how electrostatic interactions of CH_3_ with one or more H_2_O molecules influence barriers to internal rotation. Alongside works that have explored the geometrical preferences of water-containing molecular complexes, the present work contributes to knowledge of the effects of solvation on conformational flexibility. It will be shown that *V*_3_ of the CH_3_ group of 2-MI increases significantly on formation of a complex between H_2_O and 2-MI. In contrast, *V*_3_ of CH_3_ in *N*-MI⋯H_2_O (where *N*-MI is *N*-methylimidazole) is very similar to the value of this parameter in the isolated *N*-MI monomer.^9^ It will be shown that these observations are consistent with the geometries of the complexes. An electrostatic (through-space) interaction between the H_2_O molecule and CH_3_ group is present in the geometry of 2-MI⋯H_2_O but not in *N*-MI⋯H_2_O. Experimentally-determined structural parameters that define the position and orientation of the water molecule will also be reported for both the complexes examined by this work.

## 2. Experimental and theoretical methods

The microwave spectrum of each complex was recorded while probing a gaseous sample containing low concentrations of methylimidazole and water in argon. Experiments to study *N*-MI⋯H_2_O vaporised *N*-methylimidazole (Sigma Aldrich, 99%) from a reservoir, heated to a temperature of 90 °C, into a flow of argon (BOC, 99.998%) containing water vapour. Supersonic expansion ensued after injection of the gas sample into an evacuated chamber from a pulsed nozzle (Parker, Series 9) using a backing pressure of 5 bar. Separate experiments to generate and study 2-MI⋯H_2_O used a focussed laser pulse to vaporise the substance from a cylindrical target rod into the argon/H_2_O flow. The backing pressure of argon used for the experiments to study 2-MI⋯H_2_O was 7 bar. The target rod was prepared to contain 2-methylimidazole (Sigma Aldrich, 98%) and copper in a 1 : 1 ratio by mass. This rod composition was found to maximise signal intensities for transitions in the spectrum of 2-MI⋯H_2_O. The fundamental (1064 nm, 43 mJ pulse^−1^) of a Nd:YAG (Minilite II) was used for the laser vaporisation. Supersonic expansion of each gas sample led to an effective rotational temperature of approximately 3 K (estimated from the relative intensities of observed transitions). The heated reservoir, laser vaporisation source and the CP-FTMW spectrometer used by the present experiments have been described in detail elsewhere.^10–12^ Immediately prior to experiments that used either D_2_O (Sigma-Aldrich, 99.9% D atom) or H_2_^18^O (Sigma Aldrich, 97% ^18^O atom), a syringe was used to introduce a small amount of the isotopically-enriched sample into the gas line immediately before the pulsed nozzle. Typically, the introduction of 0.1 mL of isotopically-enriched sample allowed for an experiment to continue for 6 hours or longer. It was occasionally necessary to pause operation and add an additional dose of 0.1 mL in order to extend the period over which the spectrum was acquired.

The rotational spectra of *N*-MI⋯H_2_O and 2-MI⋯H_2_O were recorded between 7.0 and 18.5 GHz. The CP-FTMW spectrometer mixes a microwave pulse that linearly sweeps from 12.0 to 0.5 GHz over a duration of 1 μs against the 19 GHz reference signal of a Phase-locked Dielectric Resonant Oscillator (PDRO). Chirped pulses are generated from a 20 GS s^−1^ arbitrary waveform generator (AWG) (Tektronix AWG 7102). A low pass filter selects the 7.0–18.5 GHz sideband which is amplified by a 300 W Traveling-Wave Tube Amplifier (TWT) prior to its introduction from a horn antenna into the vacuum chamber perpendicular to the direction of the expanding gas jet. Subsequent to the molecular polarisation, the free induction decay (which has duration of 20 μs) of the molecular emission is detected by a second horn antenna and digitally recorded by a 100 GS s^−1^ oscilloscope (Tektronix DPO72304XS). Successive free induction decays are co-added in the time domain. The polarisation pulse and free induction decay are each sufficiently short that eight distinct measurements of the broadband microwave spectrum are routinely performed following each gas introduction pulse while taking advantage of the “fast frame” mode of the oscilloscope. A high resolution window function was used by the Fourier transform employed herein such that linewidths of transitions in the frequency domain spectrum are approximately 100 kHz for a well-isolated line at full-width half-maximum. This linewidth correlates with an estimated accuracy of 10 kHz in the measurement of line centre frequencies. Phase coherence in the time domain and accuracy in transition frequencies were provided by an Rb-clock (SRS FS725) to which the AWG, the PDRO and the oscilloscope were phase-locked.

Optimisations of the geometries of *N*-MI⋯H_2_O and 2-MI⋯H_2_O were performed using the Gaussian09 package.^13^ The harmonic hybrid functional^14–16^ of Becke, Lee, Yang, Parr, B3LYP, in conjunction with Grimme's dispersion correction effects^17^ and damping function,^18^ D3BJ, was initially used alongside Dunning's^19,20^ augmented triple-ζ aug-cc-pVTZ basis set. Geometry optimisations were subsequently performed with the long range corrected hybrid functional,^21^ ωB97X-D, of Chai and Gordon and Dunning's augmented quadrupole-ζ basis set with tight convergence criteria. The same level of theory was used during a previous study of imid⋯H_2_O.^4^ Structural parameters that describe the interaction between H_2_O and imidazole were assumed equal to those calculated for imid⋯H_2_O in the starting point of the geometry optimisation for each of *N*-MI⋯H_2_O and 2-MI⋯H_2_O. In each case, it was assumed that the molecule is oriented such that ∠(O–H_b_⋯N3–C2) = 0°. The *V*_3_ barrier to internal rotation was calculated by scanning the ∠(H–C6–N1–C2) or ∠(H–C6–C2–N3) dihedral angle as appropriate to each complex. The results of the geometry optimisation for each complex are shown in Table S1 (ESI†) and Fig. 1 while the calculated rotational constants (*A*_e_, *B*_e_, *C*_e_), nuclear quadrupole coupling constants, dipole moment components (|*μ*_*a*_|, |*μ*_*b*_| and |*μ*_*c*_|) and (*V*_3_) barriers to internal rotation are summarized in Table S2 (ESI†) which also shows the differences between calculated and experimentally-determined parameters. Calculations of anharmonic force fields employed the B3LYP functional with the D3BJ correction and the jun-cc-pVDZ basis set.

## 3. Results and discussion

### 3.1. Observations and spectral assignment

The most intense transitions of each methylimidazole monomer were typically observed with a S/N of at least 5 : 1 after fewer than 120 averages and were the most intense signals recorded during the described experiments. The water dimer transition at 12321 MHz was another useful reference point during initial optimisation of the spectrometer and gas expansion conditions. The DFT-calculated geometries of each of *N*-MI⋯H_2_O and 2-MI⋯H_2_O have a dipole moment that is nearly aligned with the *a* inertial axis (Fig. 1). The most intense transitions in the spectrum of each of *N*-MI⋯H_2_O and 2-MI⋯H_2_O are a-type transitions which were readily identified and assigned to the spectrum of a near-prolate symmetric rotor. Initial assignments of the molecular carriers of the observed spectra were made on basis of the agreement between the experimentally-determined and theoretically-calculated rotational constants.

A previous experimental study observed two isomers of a 1 : 1 complex formed between water and imidazole.^4^ Water was the hydrogen bond donor in the first of these isomers (denoted as imid⋯H_2_O) and the hydrogen bond acceptor in the second (denoted as H_2_O⋯imid). The bifunctional nature of the imidazole ring implies that the method employed herein might allow the generation of H_2_O⋯2-MI as well as 2-MI⋯H_2_O. Ultimately, conclusive proof that the observed spectrum should be assigned to 2-MI⋯H_2_O was obtained by measuring the shifts in rotational constants on isotopic substitution. Spectra were measured for 2-MI⋯H_2_^16^O, 2-MI⋯H_2_^18^O, 2-MI⋯DOH, 2-MI⋯HOD and 2-MI⋯D_2_O isotopologues of 2-MI⋯H_2_O. All measured isotopic shifts were highly consistent with assignment of 2-MI⋯H_2_O as the carrier of the observed spectrum. The experiments did not reveal evidence for the formation of H_2_O⋯2-MI. Beyond the spectra identified and discussed above, a number of transitions observed during the present work assign to products generated by the fragmentation of imidazole. The same range of fragmentation products was observed during previous studies^4,22^ of (imid)_2_ and imid⋯H_2_O which were performed by the same method.

**Fig. 2:**
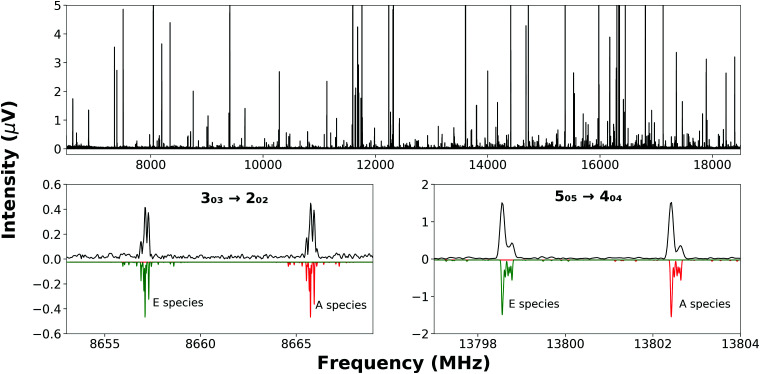
(top panel) The broadband rotational spectrum recorded while probing a sample containing 2-MI, H_2_^16^O and argon averaged over 5.1 × 10^5^ FID's. Some peaks are off-scale to allow the display of weaker features. (bottom panels) Experimentally-observed *A*- and *E*-species transitions of 2-MI⋯H_2_O are displayed (black) above a simulation (green and red) that uses the fitted values of parameters. Blending of hyperfine transitions is apparent in the experimental spectrum.

Partially resolved hyperfine structure (Fig. 2, bottom panels) was observed as consistent with the presence of two nitrogen nuclei (*I* = 1 for ^14^N) in each complex. Initially, *A*-species transition frequencies were fit with Watson's S-reduced Hamiltonian,^23^ implemented in PGOPHER;^24^1
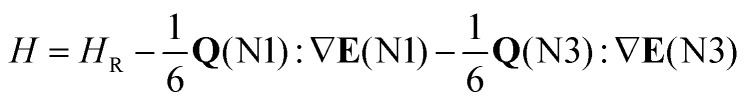
where *H*_R_ is the energy operator for a semi-rigid asymmetric rotor and the remaining terms represent interactions between the nuclear electric quadrupole moment and the electric field gradient at each nitrogen atom. *H*_R_ includes the “effective” rotational constants, 
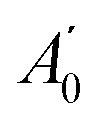
, 
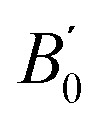
 and 
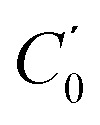
 and the centrifugal distortion terms, *D*_*J*_ and *D*_*JK*_, for both *N*-MI⋯H_2_O and 2-MI⋯H_2_O. The Hamiltonian used for *N*-MI⋯H_2_O required inclusion of an additional centrifugal distortion term, *d*_1_. The values of rotational constants and centrifugal distortion constants determined during the initial fits of spectroscopic parameters to *A*-species (*m* = 0) transition frequencies for five isotopologues of each complex are presented in Tables S3 and S4 (ESI†). The rotational constants, *A*_0_, *B*_0_ and *C*_0_ will be determined through a combined analysis of *A*- and *E*-species transitions to be presented in Section 3.2.

The nuclear quadrupole moment dyadic is represented by **Q** and the dyadic of the electric field gradient is Δ**E**. Matrix elements were constructed in the coupled asymmetric rotor basis, *J* + *I*_N1_ = *F*_1_, *F* + *I*_N3_ = *F*_2_, and diagonalised in blocks of the quantum number, *F*. Components of the nuclear quadrupole coupling tensor of each quadrupolar nitrogen nucleus are denoted by *χ*_*aa*_ and (*χ*_*bb*_ − *χ*_*cc*_) with nitrogen atoms numbered according to the convention when labelling heteroaromatic molecules with N1 and N3 denoting the pyrrolic and pyridinic nitrogen atoms respectively. Hyperfine structure was observed to be extensively blended but values of *χ*_*aa*_(N1) and *χ*_*aa*_(N3) were fitted for every isotopologue of each complex. Values of [*χ*_*bb*_(N1) − *χ*_*cc*_(N1)] for *N*-MI⋯DOH, *N*-MI⋯HOD and *N*-MI⋯D_2_O were fixed equal to the experimental result for *N*-MI⋯H_2_^16^O. Values of [*χ*_*bb*_(N3) − *χ*_*cc*_(N3)] were held fixed for most isotopologues of each complex. The procedure yielded satisfactory agreement between simulated and experimentally-observed line shapes (Fig. 2, lower panel), and between the experimentally-determined and DFT-calculated results. The electron distribution at each nitrogen atom is confirmed to be very similar to that for the corresponding atom of the imidazole monomer (Table S5, ESI†). The final cycle of each spectroscopic fit is provided within the supplementary data. The standard deviations of all fits of *A*-species transitions (Tables S3 and S4, ESI†) are between 15 and 35 kHz. Spectroscopic parameters are thus determined with high precision in spite of the line blending and assumed values of hyperfine parameters. This is consistent with previous work which found that good agreement was achieved between DFT-calculated and experimental results for *χ*_*aa*_ and (*χ*_*bb*_ − *χ*_*cc*_) for nitrogen atoms within N-heterocyclic rings.

### 3.2 Analysis of internal rotation of methyl group

It is known that there is a negligible barrier to the internal rotation of CH_3_ in toluene^25^ while the addition of substituents to the benzene ring introduces an asymmetry into molecular orbitals that leads to an increased barrier.^26–29^ The (*V*_3_) barrier to internal rotation of CH_3_ has been determined for different structural isomers of methylthiazole,^30–32^ methyloxazole^33^ and methylimidazole.^9^ The magnitude of the parameter depends on the asymmetry of the molecular orbitals with which the CH_3_ group interacts. For example, the *V*_3_ barrier to internal rotation of CH_3_ in 2-methylimidazole is smaller than that in 4- or 5-methylimidazole by a factor of three. In all of the forementioned cases, the changes in *V*_3_ result from electronic rather than steric effects. An important aim of the present work is to investigate whether the attachment of H_2_O to *N*-MI or 2-MI changes the magnitude of *V*_3_ in either isomer.

Subsequent to the identification and assignment of *E*-species transitions, combined fits of spectroscopic parameters to the frequencies of both *A*- and *E*- species transitions were performed by two different methods. Previous papers have noted that the determined value of *V*_3_ can vary depending on the Hamiltonian model employed. Fits performed in XIAM^34^ employ a Hamiltonian that is constructed in the principal axis system. XIAM treats each torsional state separately without accounting for interactions between different torsional states. The BELGI-C_s_ program^35^ employs the alternative reference framework of the rho axis method and allows the inclusion of higher-order terms. Parameters were fitted using each of XIAM and BELGI-C_s_ to explore dependencies of the results on the models employed. The experiments probed complexes in their vibrational ground states preventing the independent determination of *F*_0_ and *V*_3_. The former was therefore fixed at the value for the corresponding methylimidazole monomer in the fits performed for each of *N*-MI⋯H_2_O and 2-MI⋯H_2_O. It was also assumed that the axis of the CH_3_ rotor lies in the *ab* plane. Nuclear quadrupole coupling of multiple quadrupolar nuclei is not currently implemented by either XIAM or BELGI-C_s_ so a bespoke procedure was used to establish line centre frequencies for *A*- and *E*-species transitions. First, the rotational and centrifugal distortion constants determined by fitting *A* species transitions (those in Tables S3 and S4, ESI†) were used to determine the unsplit line centres of *A*-species rotational transitions. Next, the shift of each *E*-species transition was measured relative to its *A*-species counterpart while assisted by PGOPHER^24^ visualisation tools that aid spectral assignment. Specifically, these tools allow (i) hyperfine structure to be toggled on or off in the displayed simulation, (ii) display of a simulated spectrum which correctly mirrors the instrument resolution and (iii) simulated and experimental spectra to be overlaid and/or offset in order to match simulated features with observations. The precisely-measured interval between each *A*-species transition and its *E*-species counterpart was thus used to determine the unsplit line centre frequency for each *E*-species transition. Fits performed using XIAM determined rotational constants and centrifugal distortion constants in the principal axis system, those performed using BELGI-C_s_ determined constants in the rho axis system. The linelist used by each BELGI-C_s_ fit was the same as that used by the XIAM fit for the corresponding isotopologue. A summary of results for one isotopologue of each complex is provided in Table 1 with full details for all isotopologues provided in Tables S6–S9 (ESI†).

**Table 1:** Results of XIAM and BELGI-C_s_ fits of spectroscopic parameters to the frequencies of *A*- and *E*-species transitions. The values of *F*_0_ are fixed to the results for the methylimidazole monomers available in ref. 9. *N*_A_ and *N*_E_ denote the number of A-species and E species transitions respectively included in the fit

	*N*-MI⋯H_2_^16^O		2-MI⋯H_2_^16^O	
	XIAM	BELGI-C_s_	XIAM	BELGI-C_s_
*A*_0_ (MHz)	5010.78(21)	5011(10)^a^	4233.93(21)	4233.2(50)^a^
*B*_0_ (MHz)	1409.5628(67)	1409.5(15)^a^	1732.5621(61)	1731.00(95)^a^
*C*_0_ (MHz)	1107.2884(60)	1107.3099(43)^a^	1240.3104(66)	1238.69(18)^a^
*D*_*J*_ (kHz)	2.392(33)	3.111(49)^b^	0.233(81)	[0.233]^b^^c^
*D*_*JK*_ (kHz)	−18.57(40)	−20.6(5)^b^	7.28(78)	4.33(71)^b^
*d*_1_ (kHz)	0.820(42)	[0.959]^b^^d^	—	—
*F*_0_ (GHz)	[157.929]	[157.929]	[157.690]	[157.690]
*V*_3_ (cm^−1^)	182.23(10)	173.6(16)	154.99(8)	150.68(90)
*D*_π2*J*_(kHz)	—	—	−114(7)	—
*F*_*v*_ (MHz)	—	—	—	5.49(60)
∠(*i*, *b*) (°)	47.16(10)	46.3(3)	36.55(6)	36.56(5)
*σ*_RMS_ (kHz)	50	55	64	79
*N*_A_/*N*_E_	26/19	26/19	19/14	19/14

a Rotational constants in the principal axis system after transformation of the rho axis system inertia tensor determined by BELGI-C_s_.

b Values in the rho axis system. Centrifugal distortion constants determined by BELGI-C_s_ cannot be directly compared with those determined by XIAM because of the different models employed.

c Could not be determined by fitting and therefore fixed to the result determined by XIAM.

d Fixed to the result obtained for the *N*-MI⋯D_2_O isotopologue (Table S7, ESI).

The internal rotation parameters included in XIAM fits were the barrier to internal rotation of the CH_3_ group, *V*_3_, and the angle between the axis of the CH_3_ rotor and the *b* inertial axis which is denoted by ∠(*i*, *b*). A term which couples the methyl rotation with the overall molecular rotation and centrifugal distortion, *D*_π2*J*_, was included when fitting the data for 2-MI⋯H_2_O. The BELGI-C_s_ fits determined the off-diagonal component of the inertia tensor in the rho axis system, *D*_*AB*_, and the internal rotation parameters, *V*_3_ and ρ for each isotopologue of each complex. A term that describes the *J*(*J* + 1) dependence of the *V*_3_ barrier, *F*_V_, was also fitted for isotopologues of 2-MI⋯H_2_O. The *σ*_RMS_ of the XIAM and BELGI-C_S_ fits were higher than those obtained when fitting the *A*-species transitions in PGOPHER and higher than would typically be expected given the experimental linewidth of 100 kHz because of blending of different hyperfine components in the observed spectra and the need to estimate rotational line centre frequencies for the XIAM and BELGI-C_s_ fits.

The rotational constants and distortion constants output by BELGI-C_S_ fits are in the rho axis system so the inertia tensor is not diagonalised and rotational constants cannot be directly compared with those determined in the principal axis system. The inertia tensor determined by the BELGI-C_s_ fits was transformed into the principal axis system for the purposes of the comparison shown in rows 1–3 of Table 1. The value of *V*_3_ for the CH_3_ group of *N*-MI⋯H_2_^16^O is determined to be 182.23(10) cm^−1^ by XIAM with other isotopologues having *V*_3_ ranging from 182.08(8) cm^−1^ to 182.41(7) cm^−1^. These results are very similar to the value of this parameter for the *N*-MI monomer^9^ which was determined to be 185.104(11) cm^−1^ through fitting in XIAM. The same parameter for *N*-MI⋯H_2_^16^O is 173.6(16) cm^−1^ when fitted using BELGI-C_s_ with other isotopologues having *V*_3_ ranging from 173.2(2) cm^−1^ to 177.2(7) cm^−1^. The XIAM fits yielded a result for *V*_3_ of 154.99(8) cm^−1^ for 2-MI⋯H_2_^16^O with results spanning from 152.65(4) cm^−1^ to 156.02(12) cm^−1^ for other isotopologues. The fits performed using BELGI-C_S_ determined the parameter to be 150.68(90) cm^−1^ for 2-MI⋯H_2_^16^O with values determined for other isotopologues ranging from 152.58(11) cm^−1^ to 155.5(24) cm^−1^. The results determined for *V*_3_ of the CH_3_ group in 2-MI⋯H_2_^16^O are all significantly higher than the value of the parameter for the 2-MI monomer which was reported to be 122.7529(38) cm^−1^.^9^ The reasons for this difference will be explored further in Section 4.

Values of ∠(*i*, *b*) fitted using XIAM and BELGI-C_s_ are in excellent agreement for all isotopologues. This parameter ranges from 46.06(3) to 47.54(7)° in the fits of internal rotor parameters for *N*-MI⋯H_2_O whereas the value implied by the *r*_0_ fit of the molecular geometry (to be described in Section 3.3) is 46.1°. The values of ∠(*i*, *b*) determined in the fits of internal rotor parameters for isotopologues of 2-MI⋯H_2_O range from 34.86(3) to 36.56(5)° while the *r*_0_ fit of the molecular geometry implies a result of 35.3° for this parameter. This high level of agreement provides confidence in the results of the internal rotor fits while also justifying assumptions that will be made to determine the *r*_0_ geometries of *N*-MI⋯H_2_O and 2-MI⋯H_2_O.

### 3.3 Characterisation of molecular geometries

In the discussion that follows, H_b_ denotes a hydrogen atom of water that forms a hydrogen bond to the pyridinic nitrogen of imidazole. The free hydrogen of the water sub-unit will be labelled as H_nb_. Inertial defects calculated from the rotational constants provide the first insight into the molecular geometry and are determined as follows,

2 *Δ*_0_ = *I*_*cc*_ − *I*_*aa*_ − *I*_*bb*_

where *I*_*aa*_, *I*_*bb*_ and *I*_*cc*_ are respectively the moments of inertia about the *a*, *b* and *c* inertial axes. The inertial defects, *Δ*_0_, of *N*-MI and 2-MI were previously shown to be −3.2070(1) u Å^2^ and −3.1497(7) u Å^2^ respectively, consistent with expectations for geometries where only the hydrogen atoms of the CH_3_ group lie outside the ab plane. The *Δ*_0_ for *N*-MI⋯H_2_^16^O and 2-MI⋯H_2_^16^O are herein determined to be −2.983(6) u Å^2^ and −3.60(4) u Å^2^ respectively. These results (and those which follow in Section 3.3) were determined from the values of *A*_0_, *B*_0_ and *C*_0_ yielded by the XIAM fits which have lower uncertainties than those obtained while using BELGI-C_s_.

The small changes in *Δ*_0_ when H_2_O attaches to either *N*-MI or 2-MI thus confirm that each complex forms in a geometry where all heavy atoms lie within the *ab* plane. In general, contributions of in-plane vibrations to *Δ*_0_ are negative whereas out-of-plane vibrations make positive contributions. The magnitudes of changes in *Δ*_0_ on addition of H_2_O to each of imidazole,^4^ thiazole^6^ and isoxazole^7^ are −0.02, −0.05 and +0.2 u Å^2^ respectively which are broadly consistent with the observations of the present work. In striking contrast, the change in *Δ*_0_ when attaching H_2_O to pyridine^36^ has been observed to be greater than 8 u Å^2^. There are significant changes in *Δ*_0_ upon isotopic substitution at H_nb_ which are presented in Tables S6 and S8 (ESI†) and discussed further below.

Determination of the rotational constants for five isotopologues of each complex allows the calculation of substitution (*r*_s_) coordinates for the hydrogen and oxygen atoms of the water molecule using Kisiel's program “KRA” available from the PROSPE website.^37^ The rotational constants obtained from the XIAM fits of *A*- and *E*-species transitions were used to calculate *r*_s_ coordinates for H_b_, H_nb_ and O which are shown alongside Costain errors^38^ in Table 2. The Kraitchman method determines the magnitudes of atomic coordinates while the signs are those inferred from the results of the DFT calculations. The results unambiguously confirm that the oxygen atom lies in the *ab* plane in each complex and that the molecular geometry of each of *N*-MI⋯H_2_O and 2-MI⋯H_2_O contains a hydrogen bond between water (acting as a proton donor) and the pyridinic nitrogen.

**Table 2:** Comparison of DFT-calculated (*r*_e_) and experimentally-determined (*r*_s_) coordinates

	Method	*a*/Å	*b*/Å	*c*/Å
*N*-MI⋯H_2_O				
H_b_	*r*_e_ (calc.)^a^	2.6691	–0.1954	–0.0341
	*r*_s_ (exp.)	2.7785(8)^b^	[0]^c^	0.05(4)
O	*r*_e_ (calc.)	3.5881	–0.5061	–0.0938
	*r*_s_ (exp.)	3.4036(5)	–0.650(3)	[0]
H_nb_	r_e_ (calc.)	3.9972	–0.2262	0.7223
	r_s_ (exp.)	3.9163(7)	–0.728(4)	0.462(6)
2-MI⋯H_2_O				
H_b_	*r*_e_ (calc.)	2.1105	–0.7120	–0.0300
	*r*_s_ (exp.)	2.064(1)	–0.784(3)	0.03(7)
O	*r*_e_ (calc.)	3.0707	–0.5535	–0.0549
	*r*_s_ (exp.)	3.0709(6)	–0.538(3)	[0]
H_nb_	*r*_e_ (calc.)	3.4265	–1.0444	0.6827
	*r*_s_ (exp.)	3.5032(6)	–1.181(2)	0.359(6)

a *r*_e_ geometries are calculated at the ωB97XD/aug-cc-pVQZ level.

b Numbers in parentheses are Costain errors.^38^

c Imaginary values were obtained by the *r*_s_ method for coordinates indicated in square brackets which are assumed equal to zero.

The *r*_0_ method^39^ (as implemented within Kisiel's STRFIT program^37^), which allows a determination of structural parameters from zero-point rotational constants, was used to determine the orientation of the water molecule relative to the imidazole ring. The geometrical parameters that are internal to the *N*-MI and 2-MI monomers^9^ must first be assumed equal to their values in the DFT-calculated (*r*_e_) geometries of *N*-MI⋯H_2_O and 2-MI⋯H_2_O respectively. The experimentally-determined rotational constants (*A*_0_, *B*_0_ and *C*_0_) determined by XIAM now allow fitting of the length of the intermolecular hydrogen bond, *r*(H_b_⋯N3), and two angles which define the position and orientation of the water molecule, ∠(H_b_⋯N3–C2) and ∠(O–H_b_⋯N3), for each of *N*-MI⋯H_2_O and 2-MI⋯H_2_O. Results were determined while assuming ∠(H_nb_–O–H_b_⋯N3) = 180° and ∠(O–H_b_⋯N3–C2) = 0° for each complex. These dihedral angles lead to sets of structural parameters (shown in Table 3) that are highly consistent with the results of the DFT calculations. An alternative assumption that ∠(O–H_b_⋯N3–C2) = 180° for 2-MI⋯H_2_O would yield *r*(H_b_⋯N3) = 1.957(23) Å, ∠(H_b_⋯N3-C2) = 107.3(18)° and ∠(O-H_b_⋯N3) = 154.4(60)° which are somewhat different from the DFT-calculated results. The assumption that ∠(O–H_b_⋯N3–C2) = 180° for *N*-MI⋯H_2_O would yield ∠(O–H_b_⋯N3)= 127(6)° which is certainly not consistent with the DFT calculated results. Fits performed while assuming that ∠(H_nb_–O–H_b_⋯N3) = 0° do not converge for either complex.

**Table 3:** Comparison of DFT-calculated (*r*_e_) and experimentally-determined (*r*_0_) coordinates

Molecule	Parameter	Method	Value
*N*-MI⋯H_2_O	*r*(H_b_⋯N3)/Å	*r*_e_ (calc.)^a^	1.897
		*r*_0_ (exp.)	1.922(4)^b^
	∠(H_b_⋯N3–C2)/°	*r*_e_ (calc.)	115.7
		*r*_0_ (exp.)	101.0(16)
	∠(O–H_b_⋯N3)/°	*r*_e_ (calc.)	170.1
		*r*_0_ (exp.)	177(5)
2-MI⋯H_2_O	*r*(H_b_⋯N3)/Å	*r*_e_ (calc.)	1.8807
		*r*_0_ (exp.)	1.923(5)
	∠(H_b_⋯N3–C2)/°	*r*_e_ (calc.)	114.3
		*r*_0_ (exp.)	116.9(9)
	∠(O–H_b_⋯N3)/°	*r*_e_ (calc.)	165.4
		*r*_0_ (exp.)	166.3(28)

a *r*_e_ geometries are calculated at the *ω*B97XD/aug-cc-pVQZ level.

b Numbers in parentheses are one standard deviation in units of the final significant figure.

The DFT calculations of the present work identify that H_nb_ lies outside the plane of the imidazole ring in each of *N*-MI⋯H_2_O and 2-MI⋯H_2_O in the equilibrium (*r*_e_) geometry. Previous works^4,6^ have found that similar “free” hydrogen atoms in related complexes undergo rapid zero-point vibrational motions such that they cannot be precisely located by *r*_0_ or *r*_s_ methods. Comparing the results obtained by the *r*_s_ and *r*_e_ methods, the *a* and *b* coordinates of H_b_ and O are highly consistent. The Kraitchman method is less precise when used to calculate the position of an atom that is close to an inertial axis. *N*-MI⋯H_2_O represents a particularly challenging case because both hydrogen atoms are very near to the *a* inertial axis. Changes in zero-point vibrational motions on isotopic substitution also introduce inaccuracies and these are particularly severe when the *r*_*s*_ coordinates of a hydrogen atom are determined. These sources of inaccuracy explain small variations in the results for the *b*-coordinate of H_b_ obtained by the *r*_e_ and *r*_s_ methods for each complex. Significant variation in the |*b*| and |*c*| coordinates of H_nb_ as determined by the *r*_0_, *r*_s_ and *r*_e_ methods results from the rapid, zero-point vibrational motions mentioned earlier. Variation in the inertial defects measured for different isotopologues is informative here (Tables S6 and S8, ESI†). For each of *N*-MI⋯H_2_O and 2-MI⋯H_2_O, the *Δ*_0_ of isotopologues that contain H_2_^16^O, DOH or H_2_^18^O are very similar (within 0.02 u Å^2^ of each other) supporting the conclusion that H_b_ and O are localised within the plane of the imidazole ring in the zero-point state. However, isotopologues that are deuterated in the H_nb_ position have |*Δ*_0_| which are between 0.2 and 0.4 u Å^2^ higher than those of isotopologues which are not deuterated at H_nb_. Evidently, out-of-plane contributions to zero-point vibrational motions mean that H_nb_ is not localised to the *ab* plane on the timescale of the molecular rotation. To facilitate broader comparisons, Tables S10 and S11 (ESI†) provide atomic coordinates calculated by the *r*_0_ method.

The analysis thus far has used the experimentally-determined rotational constants for the zero-point vibrational state of each complex to determine *r*_0_ and *r*_s_ geometrical parameters. These *r*_0_ and *r*_s_ parameters cannot necessarily be expected to agree with *r*_e_ (equlibrium) parameters with very high precision because the former are influenced by zero-point vibrational motions. However, it is possible to calculate semi-experimental equilibrium rotational constants^5,40^ (*B*^*i*^_e_) from experimentally-determined rotational constants (*B*^*i*^_0_), where *i* indicates permutation over the *a*, *b*, *c* inertial axes, using3
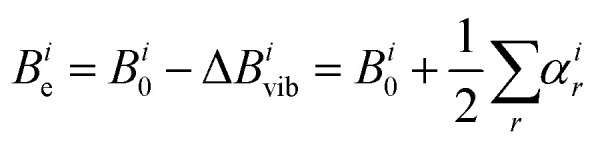
where the *α*^*i*^_r_ are vibration–rotation interaction constants determined by summing over all *r* vibrational modes. First, the anharmonic force field (and thus Δ*B*^*i*^_vib_) of the complex is computed using density functional theory. Next, the calculated value of Δ*B*^*i*^_vib_ is subtracted from the experimentally-determined value of *B*^*i*^_0_ to yield *B*^*i*^_e_ (this step is performed for each isotopologue of the complex). Finally, geometrical parameters (denoted as semi-experimental equilibrium, or *r*^SE^_e_, parameters) are fitted to the *B*^*i*^_e_ determined for the various isotopologues. Provided that the anharmonic force field has been accurately computed, the resulting *r*^SE^_e_ parameters can then be directly compared with the results of theoretical calculations that yield *r*_e_ parameters.

During the present work, calculations of anharmonic force fields to yield values of *α*^*i*^_r_ for each of *N*-MI⋯H_2_O and 2-MI⋯H_2_O were performed using the B3LYP functional, the partially-augmented double-ζ jun-cc-pVDZ basis set and Grimme's DFT-D3 scheme with the Becke–Johnson (BJ) damping function for the treatment of dispersion effects. Prior to calculation of the anharmonic force field, the geometry of the complex was re-optimised at this level of theory. This scheme for calculation of the anharmonic force field was previously used by Melli *et al* during their recent study of imid⋯H_2_O. Values of *α*^*i*^_r_ and hence the semi-experimental equilibrium rotational constants, *B*^*i*^_e_, were calculated for isotopologues of each complex as shown in Tables S12 and S13 (ESI†). A least squares fit of the values of *r*(H_b_⋯N3), ∠(H_b_⋯N3–C2) and ∠(O–H_b_⋯N3) to the semi-experimental equilibrium rotational constants (*B*^*i*^_e_) determined for the various isotopologues of 2-MI⋯H_2_O yielded values of 1.912(10) Å, 116.6(12)° and 160.8(38)° respectively for these parameters. A determination of *r*^SE^_e_ parameters for *N*-MI⋯H_2_O by the same method yielded *r*(H_b_⋯N3) = 1.904(20) Å, ∠(H_b_⋯N3–C2) = 104.0(42)° and ∠(O–H_b_⋯N3) = 168(13)°. For each complex, the *r*^SE^_e_ value of *r*(H_b_⋯N3) is in better agreement with the DFT-calculated *r*_e_ result than is the *r*_0_ parameter. However, particularly for *N*-MI⋯H_2_O, the determined *r*^SE^_e_ angles are associated with high uncertainties. The discussion of Section 4 will therefore compare values of *r*_0_ parameters determined with high precision for imid⋯H_2_O, *N*-MI⋯H_2_O and 2-MI⋯H_2_O. Differing zero-point vibrational motions within each complex mean that precise agreement should not necessarily be expected in comparisons of *r*_0_ parameters determined for different complexes.

## 4. Discussion

Both the DFT calculations and the experimental measurements confirm that a hydrogen bond forms between H_b_ and the pyridinic nitrogen of imidazole in both *N*-MI⋯H_2_O and 2-MI⋯H_2_O. The length of this hydrogen bond when determined by the *r*_0_ method is 1.922(4) Å in *N*-MI⋯H_2_O, 1.923(5) Å in 2-MI⋯H_2_O and was found to be 1.927(27) Å in the earlier study of imid⋯H_2_O. The results for these bond lengths are effectively identical given the approximations involved. The results for the determined bond angles and barriers to internal rotation provide insight into a weak electrostatic interaction between H_2_O and the neighbouring hydrogen atom or methyl group that is attached to C2 of imidazole in *N*-MI⋯H_2_O and 2-MI⋯H_2_O respectively.

Melandri *et al.*, McGlone *et al.*, Caminati *et al.*, McKenzie *et al.* and Li *et al.* identified non-linear hydrogen bonds in pyrimidine⋯H_2_O,^8^ isoxazole⋯H_2_O,^7^ pyridazine⋯H_2_O,^41^ pyrazine⋯H_2_O,^42^ pyridine⋯H_2_O^36^ and thiazole⋯H_2_O^6^ respectively. Our group recently reported that imid⋯H_2_O^4^ contains a non-linear hydrogen bond where ∠(O–H_b_⋯N3) = 172.1(26)° in the *r*_0_ geometry. In each of the studies listed above, the non-linearity of the hydrogen bond results from a weak electrostatic interaction between the oxygen of H_2_O and a hydrogen on the C2 position of the aromatic ring. The local environments at the pyridinic nitrogen atoms of imidazole and *N*-methylimidazole are very similar. Unsurprisingly, therefore, the *r*_0_ results of ∠(H_b_⋯N3–C2) = 101.1(16)° and ∠(O–H_b_⋯N3) = 177(5)° reported herein for *N*-MI⋯H_2_O are similar to the results reported for imid⋯H_2_O. The oxygen atom is a distance of 3.081(6) Å from the hydrogen attached to C2 in the *r*_0_ geometry of *N*-MI⋯H_2_O while the separation between these two atoms in imid⋯H_2_O is 3.13(8) Å. The separation between oxygen and the hydrogen atoms of the CH_3_ in 2-MI⋯H_2_O depends on the value of the dihedral angle, ∠(N3–C2–C6–H), which is scanned by the CH_3_ internal rotation. When ∠(N3–C2–C6–H) = 0°, the separation between oxygen and the nearest hydrogen is 2.566(3) Å while the assumption that ∠(N3–C2–C6–H) = 60° places two hydrogen atoms only 3.175(3) Å from the oxygen atom. Evidently, oxygen interacts with its neighbouring hydrogen atoms over shorter distances (on average) within 2-MI⋯H_2_O than within imid⋯H_2_O or *N*-MI⋯H_2_O. These shorter interaction distances lead to ∠(O–H_b_⋯N3) = 166.3(28)° and hence enhanced non-linearity of the primary hydrogen bond in 2-MI⋯H_2_O relative to either imid⋯H_2_O or *N*-MI⋯H_2_O. The hydrogen bond^7^ in isoxazole⋯H_2_O was reported to be significantly longer, and less linear, than those identified in imid⋯H_2_O, *N*-MI⋯H_2_O and 2-MI⋯H_2_O (Table 4).

**Table 4:** Comparison of experimentally-determined (*r*_0_) structural parameters for complexes^4,7^ formed between 5-membered N-heterocyclic rings and H_2_O

	*r*(H_b_⋯N3)/Å	∠(O–H_b_⋯N3)/°
Imidazole⋯H_2_O	1.927(27)^a^	174.7(24)
*N*-Methylimidazole⋯H_2_O	1.922(4)	177(5)
2-Methylimidazole⋯H_2_O	1.923(5)	166.3(28)
Isoxazole⋯H_2_O	2.1467	141.12

a Uncertainties in parentheses are those quoted in the primary source.

Barriers to internal rotation have been extensively explored for CH_3_ substituents of aromatic rings. Where there is two-fold symmetry about the axis of a CH_3_ rotor, electronic overlap between a π-like orbital on the CH_3_ and π-orbitals on the ring leads to a very low barrier to internal rotation.^43^ The magnitude of the barrier increases with the asymmetry of participating molecular orbitals such that it is significantly higher for 4- or 5-methylimidazole than for 2-methylimidazole.^9^ The same trend is apparent in published results for the 2-, 4- and 5- isomers of methylthiazole^30–32^ and methyloxazole.^33^ The value for *V*_3_ of the *N*-methylimidazole monomer^9^ (determined using XIAM) is 185.104(11) cm^−1^ which is very similar to the results of 182.21(12) cm^−1^ and 173.6(16) cm^−1^ for *N*-MI⋯H_2_^16^O determined through fits performed using XIAM and BELGI-C_s_ respectively. The water molecule is on the opposite side of the molecule so does not interact through space with the CH_3_ group within *N*-MI⋯H_2_O. The evidence of the experiments reported herein is that the attachment of H_2_O to *N*-MI does not significantly change the electronic environment at the CH_3_ group either. The results for *N*-MI⋯H_2_O are thus consistent with previous studies where (*V*_3_) barriers to internal rotation of CH_3_ groups were not observed to change in response to the attachment of H_2_O to a remote site of the molecule.

The *V*_3_ barrier determined for 2-MI⋯H_2_^16^O (determined to be 154.99(8) cm^−1^ by XIAM and 150.68(90) cm^−1^ by BELGI-C_s_) is significantly greater than the value determined for the 2-MI monomer^9^ by XIAM (122.7529(38) cm^−1^). An important question is whether this difference arises primarily because of electronic or steric effects. Does the attachment of H_2_O induce changes in the symmetry of molecular orbitals on the imidazole ring? Alternatively, is the presence of a weak electrostatic interaction between the oxygen atom and the CH_3_ group of 2-MI sufficient to explain the change in *V*_3_ that follows the attachment of H_2_O? It is impossible to exclude a contribution for electronic effects but these did not lead to a significant change in *V*_3_ on attachment of H_2_O to *N*-MI. It is therefore more likely that the weak electrostatic interaction between the oxygen atom and the CH_3_ group is the predominant cause of the difference between the *V*_3_ determined for 2-MI and 2-MI⋯H_2_O. It is noted that an earlier analysis^44^ of the microwave spectrum of the toluene⋯SO_2_ complex also observed a large increase (from 4.9 cm^−1^ in free toluene to 87.3 cm^−1^ in toluene⋯SO_2_) in the barrier to internal rotation of the CH_3_ group on formation of that complex. The authors of that study modelled electrostatic interactions between SO_2_ and the CH_3_ group and found these to be sufficient to explain the observed change in the barrier. The authors did not exclude the possibility that electronic effects also contribute.

## Conclusions

Each of *N*-MI⋯H_2_O and 2-MI⋯H_2_O contains a primary hydrogen bond between the hydrogen of H_2_O and the pyridinic nitrogen of the imidazole ring. The length of the primary hydrogen bond is very similar in *N*-MI⋯H_2_O, 2-MI⋯H_2_O and imid⋯H_2_O. A weak electrostatic interaction between the oxygen atom of H_2_O and hydrogens (or the CH_3_ group) attached to the C2 position of the imidazole ring leads to a non-linear hydrogen bond being present in each of *N*-MI⋯H_2_O and 2-MI⋯H_2_O. The (*V*_3_) barrier to internal rotation of the CH_3_ group of *N*-MI⋯H_2_O is essentially unchanged from that observed for the *N*-MI monomer. In contrast, the (*V*_3_) barrier to internal rotation of the CH_3_ group within the 2-MI⋯H_2_O complex is significantly higher than that of the CH_3_ group within the 2-MI monomer because of the weak interaction between the oxygen atom of water and the CH_3_ group.

## Author contributions

Medcraft and Heitkämper contributed to investigation. Gougoula, Cummings contributed to conceptualisation, formal analysis, investigation, validation and writing. Walker contributed to supervision, funding acquisition and writing.

## Conflicts of interest

There are no conflicts to declare.

## Supplementary Material

CP-024-D1CP05526G-s001

CP-024-D1CP05526G-s002

CP-024-D1CP05526G-s003

CP-024-D1CP05526G-s004

CP-024-D1CP05526G-s005

CP-024-D1CP05526G-s006

CP-024-D1CP05526G-s007

CP-024-D1CP05526G-s008

CP-024-D1CP05526G-s009

CP-024-D1CP05526G-s010

CP-024-D1CP05526G-s011

CP-024-D1CP05526G-s012

CP-024-D1CP05526G-s013

CP-024-D1CP05526G-s014

CP-024-D1CP05526G-s015

CP-024-D1CP05526G-s016

CP-024-D1CP05526G-s017

CP-024-D1CP05526G-s018

CP-024-D1CP05526G-s019

CP-024-D1CP05526G-s020

CP-024-D1CP05526G-s021

CP-024-D1CP05526G-s022

CP-024-D1CP05526G-s023

CP-024-D1CP05526G-s024

CP-024-D1CP05526G-s025

CP-024-D1CP05526G-s026

CP-024-D1CP05526G-s027

CP-024-D1CP05526G-s028

CP-024-D1CP05526G-s029

CP-024-D1CP05526G-s030

CP-024-D1CP05526G-s031
