# Supplementary material for: Microwave spectra, molecular geometries, and internal rotation of CH3 in N-methylimidazole⋯H2O and 2-methylimidazole⋯H2O Complexes
Source: Phys Chem Chem Phys. 2022 May 6;24(20):12354–62. doi: 10.1039/d1cp05526g (PMC9131724; doi:10.1039/d1cp05526g)
Supplement: CP-024-D1CP05526G-s025 [file CP-024-D1CP05526G-s025.pdf]

Electronic Supplementary Material (ESI) for Physical Chemistry Chemical Physics.
This journal is © the Owner Societies 2022

Phys. Chem. Chem. Phys.

Supplementary Information

Microwave Spectra, Molecular Geometries, and Internal Rotation of CH_3 in *N*-methylimidazole…H_2O and 2-methylimidazole…H_2O Complexes

Eva Gougoula,[a] Charlotte N. Cummings,[b] Chris Medcraft,[c] Juliane Heitkämper,[b] and Nicholas R. Walker[b]*

[a] Deutsche Elektronen-Synchrotron (DESY), Notkestraße 85, D-22607 Hamburg.

[b] Chemistry- School of Natural and Environmental Sciences, Newcastle University, Bedson Building, Newcastle-upon-Tyne, NE1 7RU, UK

[c] School of Chemistry, UNSW Sydney, Sydney, New South Wales 2052, Australia.

KEYWORDS: rotational spectroscopy, internal rotation, imidazole, chirped-pulse, microwave.

AUTHOR INFORMATION

Corresponding Author

*nick.walker@ncl.ac.uk

Table S1 – Atomic coordinates in the optimised geometries of *N*-MI…H_2O and 2-MI…H_2O calculated at the ωB97X-D/aug-cc-pVQZ level.

N-MI…H_2O			
	a/Å	*b*/Å	*c*/Å
N(1)	–1.357174	–0.231787	0.008588
C(2)	–0.099160	–0.721263	0.062897
H(2)	0.107524	–1.778352	0.101293
N(3)	0.796658	0.232451	-0.061732
C(4)	0.087957	1.400243	0.001122
H(4)	0.576279	2.358912	–0.015310
C(5)	–1.247427	1.133689	–0.033036
H(5)	–2.110130	1.773649	–0.079802
C(6)	–2.581119	–0.998193	–0.035331
H(6)	–2.347795	–2.048243	0.119485
H(6)	–3.260370	–0.673033	0.750364
H(6)	–3.070516	–0.885539	–1.001446
H_b	2.669123	–0.195381	–0.034050
O	3.588113	–0.506146	–0.093761
H_{nb}	3.997168	–0.226173	0.722259
2-MI…H_2O			
	a/Å	*b*/Å	*c*/Å
N(1)	1.610674	–0.633079	–0.001090
H(1)	2.201624	–1.442578	–0.005724
C(2)	0.254852	–0.642298	0.012642
N(3)	–0.207781	0.583735	0.014195
C(4)	0.886215	1.411753	–0.001147
H(4)	0.782666	2.482594	–0.005503
C(5)	2.027401	0.675993	–0.010290
H(5)	3.066282	0.949231	–0.020542
C(6)	–0.560578	–1.885871	0.004610
H(6)	–1.606181	–1.625183	0.146866
H(6)	–0.463607	–2.409208	–0.947316
H(6)	–0.254048	–2.566814	0.798947
H_b	–2.110527	0.712036	–0.029976
O	–3.070668	0.553547	–0.054869
H_{nb}	–3.426471	1.044396	0.682725

Table S2 – Spectroscopic parameters of *N*-MI…H_2O and 2-MI…H_2O calculated at different levels of theory. The calculated rotational constants are appropriate to the equilibrium geometry whereas experimental results are for the zero-point state.

N-MI…H_2O				
	ωB97X-D/aug-cc-pVQZ		B3LYP(D3BJ)/aug-cc-pVTZ	
A_e (MHz)	6111	+22 %[a]	5744	+15 %[a]
B_e (MHz)	1277	–9 %	1314	–7 %
C_e (MHz)	1067	–4 %	1080	–3 %
$\chi_{aa}(N1)$ (MHz)	1.37	+23 %	1.38	+24 %
$[\chi_{bb}(N1) - \chi_{cc}(N1)]$ (MHz)	3.69	0 %	3.86	+5 %
$\chi_{aa}(N3)$ (MHz)	–4.00	+8 %	–3.94	+7 %
$[\chi_{bb}(N3) - \chi_{cc}(N3)]$ (MHz)	–0.40	-[b]	–0.14	-[b]
V_3 (cm^{-1})[c]	230	+21 %	185	+2 %
$\lvert\mu_a\rvert$, $\lvert\mu_b\rvert$, $\lvert\mu_c\rvert$ (D)[b]	6.1, 0.1, 1.2		5.9, 0, 1.2	
**2-MI…H_2O**				
	ωB97X-D/aug-cc-pVQZ		B3LYP(D3BJ)/aug-cc-pVTZ	
A_e (MHz)	4198	–1 %	4183	–1 %
B_e (MHz)	1759	2 %	1769	+2 %
C_e (MHz)	1252	1 %	1256	+1 %
$\chi_{aa}(N1)$ (MHz)	1.24	–9 %	1.31	–4 %
$[\chi_{bb}(N1) - \chi_{cc}(N1)]$ (MHz)	4.18	+1 %	4.24	+2 %
$\chi_{aa}(N3)$ (MHz)	–3.37	4 %	–3.27	+1 %
$[\chi_{bb}(N3) - \chi_{cc}(N3)]$ (MHz)	–0.64	–4 %	–0.44	–35 %
V_3 (cm^{-1})[c]	223	+30 %	173	+11 %
$\lvert\mu_a\rvert$, $\lvert\mu_b\rvert$, $\lvert\mu_c\rvert$ (D)[d]	4.5, 1.8, 1.1		4.4, 1.6, 1.0	

[a] Calculated as [((6111.37 MHz–5010.77) / 5010.77 MHz) × 100] where 6111.37 MHz is the calculated A_e shown above, 5010.82 MHz is the result for A_0 displayed in Table 1. A similar calculation is performed for percentages in other cells.

[b] Experimental results not available for $[\chi_{bb}(N3) - \chi_{cc}(N3)]$ of n-MI…H_2O.

[c] The V_3 barrier to internal rotation was calculated by scanning the ∠(H–C6–N1–C2) or ∠(H–C6–C2–N3) dihedral angle as appropriate to each complex.

[d] Electric dipole moment components along the principal inertial axes.

Table S3 – Spectroscopic parameters determined by fitting *A*-species transitions of *N*-MI…H_2O.

	$H_2{}^{16}O$	$H_2{}^{18}O$	DOH	HOD	D_2O
A_0' (MHz)	5016.647(83)[a]	4979.14(17)	5036.72(11)	4981.73(38)	5002.87(11)
B_0' (MHz)	1409.9245(26)	1325.5593(63)	1380.3348(32)	1351.4172(39)	1324.5280(39)
C_0' (MHz)	1107.2933(23)	1052.8779(42)	1089.9289(26)	1070.2048(39)	1054.3099(28)
D_J (kHz)	2.393(12)	2.152(33)	2.271(17)	2.065(64)	1.963(18)
D_{JK} (kHz)	–19.24(12)	–16.53(39)	–18.37(25)	–12.0(26)	–14.16(19)
d_1 (kHz)	–0.821(16)	–0.733(27)	–0.808(18)	[–0.821]	–0.692(17)
$\chi_{aa}(N1)$ (MHz)	1.102(53)	1.23(82)	1.40(19)	1.6(11)	0.89(36)
$[\chi_{bb}(N1) - \chi_{cc}(N1)]$ (MHz)	3.69(26)	3.33(80)	[3.69][b]	[3.69][b]	[3.69][b]
$\chi_{aa}(N3)$ (MHz)	–3.694(43)	–3.67(22)	–3.81(26)	–3.99(76)	–3.65(13)
$[\chi_{bb}(N3) - \chi_{cc}(N3)]$ (MHz)	[–0.40][c]	[–0.40][c]	[–0.40][c]	[–0.40][c]	[–0.40][c]
σ_{RMS} (kHz)	22	32	19	28	20
N	66	33	23	17	26

[a] Numbers in parentheses are one standard deviation in units of the last significant figure.

[b] Fixed equal to experimental result for n-MI…$H_2{}^{16}O$.

[c] $[\chi_{bb}(N3) - \chi_{cc}(N3)]$ fixed at result of ωB97X-D/aug-cc-pVQZ calculation (Table S2) which is –0.40 MHz.

Table S4– Spectroscopic parameters determined by fitting *A*-species transitions of 2-MI…H_2O

	$H_2{}^{16}O$	$H_2{}^{18}O$	DOH	HOD	D_2O
A_0' (MHz)	4238.327(41)[a]	4219.869(44)	4216.661(46)	4188.097(48)	4169.345(36)
B_0' (MHz)	1733.7666(11)	1629.8925(14)	1708.7911(18)	1662.8599(17)	1640.2022(13)
C_0' (MHz)	1240.3151(12)	1184.7889(18)	1225.7117(17)	1200.1960(18)	1186.9046(16)
D_J (kHz)	0.189(16)	0.235(18)	0.249(17)	0.322(13)	0.277(14)
D_{JK} (kHz)	7.45(15)	7.81(18)	6.07(35)	6.90(17)	6.52(14)
$\chi_{aa}(N1)$ (MHz)	1.312(35)	1.390(67)	1.293(61)	1.258(76)	1.362(87)
$[\chi_{bb}(N1) - \chi_{cc}(N1)]$ (MHz)	4.11(10)	3.92(26)	3.98(21)	3.79(21)	4.14(26)
$\chi_{aa}(N3)$ (MHz)	–3.282(78)	–3.12(10)	–3.120(62)	–2.991(59)	–3.247(67)
$[\chi_{bb}(N3) - \chi_{cc}(N3)]$ (MHz)	[–0.67][b]	[–0.67][b]	[–0.67][b]	[–0.67][b]	–0.67(18)
σ_{RMS} (kHz)	18	27	23	30	24
N	72	65	40	60	76

[a] Numbers in parentheses are one standard deviation in units of the last significant figure.

[b] The value of $[\chi_{bb}(N3) - \chi_{cc}(N3)]$ of 2-MI…D_2O is consistent with the calculated result (Table S3) and determined with reasonable precision. Values of $[\chi_{bb}(N3) - \chi_{cc}(N3)]$ for all other isotopologues were not determined by the fits performed. For these reasons, $[\chi_{bb}(N3) - \chi_{cc}(N3)]$ for 2-MI…H_2O, 2-MI…$H_2{}^{18}O$, 2-MI…DOH, 2-MI…HOD were fixed equal to the result for 2-MI…D_2O.

Table S5 – Nuclear quadrupole coupling constants in the principal nuclear axis framework. The ωB97X-D/aug-cc-pVQZ results for *N*-MI…H_2O and 2-MI…H_2O are determined from the results for χ_{aa} and $[\chi_{bb} - \chi_{cc}]$ provided in Table S2. The results provided for imidazole are from D. Christen *et al.*, Z. Naturforsch. 1982, 37a, 1378.

	ωB97X-D/aug-cc-pVQZ		Exp. (FTMW)
	N-MI…H_2O	2-MI…H_2O	Imidazole
$\chi_{xx}(N1)$ (MHz)	0.8600[a]	1.1705[a]	0.890(52)
$\chi_{yy}(N1)$ (MHz)	1.6797	1.5385	1.633(58)
$\chi_{zz}(N1)$ (MHz)	–2.5397	–2.7091	–2.524(12)
$\chi_{xx}(N3)$ (MHz)	1.8256	2.0059	1.835(24)
$\chi_{yy}(N3)$ (MHz)	2.2058	1.8991	2.278(24)
$\chi_{zz}(N3)$ (MHz)	–4.0314	–3.9050	–4.113(34)

a. Calculated using the QDIAG program available on the PROSPE website < http://www.ifpan.edu.pl/~kisiel/prospe.htm>.

Table S6 Results of global (XIAM) fit of spectroscopic parameters to the frequencies of *A*- and *E*- species transitions of n-MI…H_2O. Numbers in parentheses are one standard deviation in units of the last significant figure. The ordering of atoms within the 3rd and 4th column headers is H_bOH_{nb} such that H_b is deuterium for the isotopologue denoted as "DOH". The value of F_0 (157.929 MHz) is fixed to the result for the *N*-MI monomer available in ref. 9. N_A and N_E denote the number of A-species and E species transitions respectively included in the fit.

	$H_2{}^{16}O$	$H_2{}^{18}O$	DOH	HOD	D_2O
A_0 (MHz)	5010.78(21)	4973.91(16)	5030.91(18)	4976.11(29)	4997.19(16)
B_0 (MHz)	1409.5628(67)	1325.2392(47)	1379.9953(53)	1351. 0759(86)	1324.2123(47)
C_0 (MHz)	1107.2884(60)	1052.8660(35)	1089.9214(47)	1070.1983(53)	1054.3030(38)
D_J (kHz)	2.392(33)	2.179(21)	2.300(26)	2.024(61)	1.980(21)
D_{JK} (kHz)	-18.57(40)	–17.02(40)	–18.78(40)	–10.9(18)	–14.14(32)
d_1 (kHz)	0.820(42)	0.780(22)	0.832(31)	0.802(48)	0.721(21)
F_0 (GHz)	[157.929]	[157.929]	[157.929]	[157.929]	[157.929]
V_3 (cm^{-1})	182.23(10)	182.41(7)	182.29(8)	182.08(8)	182.37(8)
$\angle(i, b)$ (°)	47.16(10)	46.52(7)	47.54(7)	46.81(8)	47.17(8)
Δ_0 (u Å^2)	–2.983(6)	–3.011(4)	–2.989(4)	–3.388(7)	–3.429(4)
σ_{RMS} (kHz)	50	38	31	21	36
N_A / N_E	26 / 19	24 / 22	15/12	11/8	19/19

Table S7 Results of global (BELGI-C_s) fit of spectroscopic parameters to the frequencies of *A*- and *E*- species transitions of *N*-MI…H_2O. Rotational and centrifugal distortion constants calculated by XIAM and BELGI-C_S are not directly comparable because the latter are calculated in the rho axis system. Numbers in parentheses are one standard deviation in units of the last significant figure. The ordering of atoms within the 3rd and 4th column headers is H_bOH_{nb} such that H_b is deuterium for the isotopologue denoted as "DOH". The value of F_0 of 157.929 GHz is fixed to the result for the n-MI monomer available in ref 9. N_A and N_E denote the number of A-species and E species transitions respectively included in the fit.

	$H_2{}^{16}O$	$H_2{}^{18}O$	DOH	HOD	D_2O
A_0 (MHz)	4768.6(59)	4747.95(86)	4800.7(54)	4753.9(24)	4779.6(21)
B_0 (MHz)	1651.9(60)	1550.91(87)	1610.1(55)	1573.1(24)	1542.0(21)
C_0 (MHz)	1107.3099(43)	1052.8743(24)	1089.9435(40)	1070.1941(27)	1054.2981(27)
D_J (kHz)	3.111(49)	2.708(25)	2.974(56)	2.369(42)	2.482(20)
D_{JK} (kHz)	–20.6(5)	–18.1(4)	–20.1(7)	–10.8(11)	–15.5(3)
d_1 (kHz)	[0.959]	[0.959]	[0.959]	[0.959]	0.959(17)
F_0 (GHz)	[157.929]	[157.929]	[157.929]	[157.929]	[157.929]
V_3 (cm^{-1})	173.6(16)	175.49(2)	173.2(2)	177.1(7)	174.6(7)
D_{AB} (MHz)	–902(10)	–878.9(16)	–887.2(99)	–869.2(44)	–867.4(41)
ρ (unitless)	0.0226(6)	[0.0228]	0.0227(6)	0.0237(3)	0.0228(3)
∠(*i*, *b*) (°)	46.3(3)	46.06(3)	46.6(4)	46.7(3)	46.6(3)
σ_{RMS} (kHz)	55	27	36	14	23
N_A / N_E	26 / 19	24 / 22	15/12	11/8	19/19

Table S8 Results of global (XIAM) fit of spectroscopic parameters to the frequencies of *A*- and *E*- species transitions of 2-MI…H_2O. Numbers in parentheses are one standard deviation in units of the last significant figure. The ordering of atoms within the 3rd and 4th column headers is H_bOH_{nb} such that H_b is deuterium for the isotopologue denoted as "DOH". The value of F_0 (157.690 MHz) is fixed to the result for the 2-MI monomer available in ref 9. N_A and N_E denote the number of A-species and E species transitions respectively included in the fit.

	$H_2{}^{16}O$	$H_2{}^{18}O$	DOH	HOD	D_2O
A_0 (MHz)	4233.93(21)	4216.088(93)	4212.776(60)	4184.178(58)	4165.477(74)
B_0 (MHz)	1732.5621(61)	1628.8335(43)	1707.6301(27)	1661.6617(20)	1639.0453(25)
C_0 (MHz)	1240.3104(66)	1184.7799(35)	1225.7031(25)	1200.1864(24)	1186.8983(28)
D_J (kHz)	0.233(81)	0.248(33)	0.250(28)	0.295(24)	0.246(27)
D_{JK} (kHz)	7.28(78)	7.51(37)	6.11(60)	6.70(29)	6.61(35)
F_0 (GHz)	[157.690]	[157.690]	[157.690]	[157.690]	[157.690]
V_3 (cm^{-1})	154.99(8)	156.02(12)	156.07(4)	152.65(4)	153.57(4)
$D_{\pi 2J}$ (kHz)	–114(7)	–131(4)	–126(3)	–128(2)	–131(3)
$\angle(i, b)$ (°)	36.55(6)	36.07(8)	36.20(6)	35.30(3)	34.86(3)
Δ_0 (u Å^2)	–3.60(3)	–3.580(3)	–3.599(2)	–3.840(2)	–3.865(2)
σ_{RMS} (kHz)	64	34	25	25	33
N_A / N_E	19 / 14	21 / 10	20 / 8	21 / 8	21 / 13

Table S9 Results of global (BELGI-C_s) fit of spectroscopic parameters to the frequencies of *A*- and *E*- species transitions of 2-MI…H_2O. Rotational and centrifugal distortion constants calculated by XIAM and BELGI-C_S are not directly comparable because the latter are calculated in the rho axis system. Numbers in parentheses are one standard deviation in units of the last significant figure. The ordering of atoms within the 3rd and 4th column headers is H_bOH_{nb} such that H_b is deuterium for the isotopologue denoted as "DOH". The value of F_0 of 157.690 GHz is fixed to the result for the 2-MI monomer available in ref 9. N_A and N_E denote the number of A-species and E species transitions respectively included in the fit.

	$H_2{}^{16}O$	$H_2{}^{18}O$	DOH	HOD	D_2O
A_0 (MHz)	3649.9(35)	3646.5(58)	3630.8(50)	3649.9(35)	3574.2(95)
B_0 (MHz)	2314.3(35)	2196.0(59)	2287.2(49)	2314.3(35)	2228.4(93)
C_0 (MHz)	1238.69(17)	1183.21(11)	1224.4359(22)	1238.69(17)	1185.74(22)
D_J (kHz)	[0.233]	[0.248]	[0.250]	[0.295]	[0.246]
D_{JK} (kHz)	4.33(71)	4.77(74)	2.11(55)	3.09(87)	2.87(80)
F_0 (GHz)	[157.690]	[157.690]	[157.690]	[157.690]	[157.690]
V_3 (cm^{-1})	150.68(89)	152.58(11)	155.5(24)	153.4(30)	155.5(23)
D_{ab} (MHz)	–1058.0(23)	–1071.6(39)	–1057.3(32)	–1068.4(62)	–1069.3(59)
F_v (MHz)	5.49(60)	5.36(40)	4.35(73)	4.07(96)	3.92(76)
ρ (unitless)	0.01799(28)	[0.0178]	0.01890(7)	0.01883(94)	0.01898(76)
∠(*i*, *b*) (°)	36.56(5)	36.03(14)	36.41(6)	35.66(11)	35.46(11)
σ_{RMS} (kHz)	79	79	42	84	76
N_A / N_E	19/14	21/10	20/8	21/8	21/13

Table S10 – DFT calculated (r_e) and experimentally-determined (r_0, r_s) atomic coordinates of H_2O in the complexes *N*-MI…H_2O and 2-MI…H_2O

	Method	a/Å	b/Å	c/Å
		N-MI…H_2O		
	Method	a/Å	b/Å	c/Å
	r_e (calc.)	2.6691	–0.1954	–0.0341
H_b	r_s (exp.)	2.7785(8)[b]	[0][c]	0.05(4)
	r_0 (exp.)	2.565(20)[b]	–0.21(5)	[0]
	r_e (calc.)	3.5881	–0.5061	–0.0938
O	r_s (exp.)	3.4036(5)	–0.650(3)	[0]
	r_0 (exp.)	3.430(5)	–0.6229(7)	[0]
	r_e (calc.)	3.9972	–0.2262	0.7223
H_{nb}	r_s (exp.)	3.9163(7)	–0.728(4)	0.462(6)
	r_0 (exp.)	4.05(4)	–0.11(4)	[0]
		2-MI…H_2O		
	Method	a/Å	b/Å	c/Å
H_b	r_e (calc.)	2.1105	–0.7120	–0.0300
	r_s (exp.)	2.064(1)	–0.784(3)	0.03(7)
	r_0 (exp.)	2.143(3)	–0.69(3)	[0]
	r_e (calc.)	3.0707	–0.5535	–0.0549
O	r_s (exp.)	3.0709(6)	–0.538(3)	[0]
	r_0 (exp.)	3.088(3)	–0.5219(11)	[0]
H_{nb}	r_e (calc.)	3.4265	–1.0444	0.6827
	r_s (exp.)	3.5032(6)	–1.181(2)	0.359(6)
	r_0 (exp.)	3.48(3)	–1.396(14)	[0]

[a.] r_e geometries are calculated at the ωB97XD/aug-cc-pVQZ level.

[b] Numbers in parentheses are Costain errors for r_s results and one standard deviation in units of the last significant figure for r_0 results.

[c.] Imaginary values were obtained for those r_s coordinates indicated in square brackets which are assumed equal to zero.

[d.] The assumed values of the dihedral angles, $\angle(H_b \ldots N3–C2–N1) = 180°$, $\angle(O–H_b \ldots N3–C2) = 0°$ and $\angle(H_{nb}–O–H_b \ldots N3) = 180°$ lead to $|c| = 0$ for H_b, O and H_{nb} of each complex.

Table S11 –Atomic coordinates of *N*-MI...H_2O and 2-MI...H_2O determined by the r_0 method while assuming that ∠(H_{nb}–O–H_b...N3) = 180° and ∠(O–H_b...N3–C2) = 0° for each complex (see text for details).

N-MI...H_2O			
	a/Å	*b*/Å	*c*/Å
N(1)	-1.26246(97)[a]	-0.30300(121)	0.00001(0)
C(2)	0.04860(161)	-0.57609(195)	0.00105(0)
H(2)	0.49485(396)	-1.55826(303)	0.00237(1)
N(3)	0.79263(115)	0.53411(375)	0.00185(0)
C(4)	-0.11852(361)	1.56402(155)	0.00028(0)
H(4)	0.18821(609)	2.59694(229)	-0.00055(1)
C(5)	-1.41824(239)	1.05856(159)	-0.00148(0)
H(5)	-2.28981(392)	1.69380(369)	-0.00267(1)
C(6)	-2.30775(336)	-1.31081(374)	-0.00030(0)
H(6)	-2.93233(310)	-1.19984(525)	-0.88669(0)
H(6)	-1.86523(576)	-2.30690(267)	-0.00933(1)
H(6)	-2.92152(312)	-1.21160(521)	0.89499(0)
H_b	2.56475(1963)	-0.21032(5031)	-0.00010(4)
O	3.43023(487)	-0.62292(66)	-0.00112(0)
H_{nb}	4.04639(3856)	0.11168(3688)	-0.00066(0)
2-MI...H_2O			
	a/Å	*b*/Å	*c*/Å
N(1)	-1.65447(94)	-0.62524(175)	-0.00077(0)
H(1)	-2.25903(221)	-1.41928(272)	0.00017(0)
C(2)	-0.29042(98)	-0.65153(46)	-0.00002(0)
N(3)	0.22479(102)	0.55671(129)	0.00174(0)
C(4)	-0.86404(238)	1.40814(47)	0.00116(0)
H(4)	-0.73153(411)	2.47745(26)	0.00452(0)
C(5)	-2.02952(124)	0.69991(235)	-0.00185(0)
H(5)	-3.06368(173)	1.00598(402)	-0.00134(0)
C(6)	0.57333(294)	-1.87203(186)	0.00078(0)
H(6)	0.37230(391)	-2.47159(153)	-0.88703(0)
H(6)	1.62476(247)	-1.58465(356)	0.00294(1)
H(6)	0.36899(390)	-2.47277(153)	0.88704(0)
H_b	2.14337(298)	0.68539(3005)	0.00000(5)
O	3.08813(262)	0.52193(108)	-0.00129(0)
H_{nb}	3.48294(2605)	1.39567(1369)	-0.00004(5)

[a] Results are output from fit performed using Kisiel's STRFIT. Numbers in parentheses are one standard deviation in units of the last significant figure.

Table S12 – Computed values of ΔB_{vib}^{i} and B_e^i for *N*-MI...H_2O.

See text for definitions of B_0^i, ΔB_{vib}^{i} and B_e^i. Values of B_0^i rotational constants are as shown in Table S6 (*a*, *b* and *c* denote the respective inertial axes). Values of ΔB_{vib}^{i} are computed by density functional theory using the B3LYP functional, the partially-augmented double-ζ jun-cc-pVDZ basis set and Grimme's DFT-D3 scheme with the Becke-Johnson (BJ) damping function for the treatment of dispersion effects. Values of B_e^i are determined by subtracting ΔB_{vib}^{i} from B_0^i according to the equation shown (Eqn 3 in the text).

$$B_e^i = B_0^i - \Delta B_{vib}^{i} = B_0^i + \frac{1}{2}\sum_r \alpha_r^i$$

	N-MI...$H_2{}^{16}O$			*N* -MI...$H_2{}^{18}O$		
	a	*b*	*c*	*a*	*b*	*c*
B_0^i	5010.78(21)	1409.5628(67)	1107.2884(60)	4973.91(16)	1325.2392(47)	1052.8660(35)
$\sum_r \alpha_r^i$	-48.863	20.67419	19.17657	-57.417	20.42193	18.62950
$-\Delta B_{vib}^{i}$	-24.431	10.33710	9.58829	-28.709	10.21097	9.31475
B_e^i	4986.34(21)	1419.8999(67)	1116.8767(60)	4945.20(16)	1335.4502(47)	1062.1808(35)
	N-MI...DOH			*N*-MI...HOD		
	A	*b*	*c*	*a*	*b*	*c*
B_0^i	5030.91(18)	1379.9953(53)	1089.9214(47)	4976.11(29)	1351.0759(86)	1070.1983(53)
$\sum_r \alpha_r^i$	54.382	8.29807	14.28911	18.336	12.29522	15.52354
$-\Delta B_{vib}^{i}$	27.191	4.14904	7.14456	9.168	6.14761	7.76177
B_e^i	5058.10(18)	1384.1443(53)	1097.0660(47)	4985.28(29)	1357.2235(86)	1077.9601(53)
	N-MI...D_2O					
	a	*b*	*c*			
B_0^i	4997.19(16)	1324.2123(47)	1054.3030(38)			
$\sum_r \alpha_r^i$	36.355	9.26088	14.21856			
$-\Delta B_{vib}^{i}$	18.178	4.63044	7.10928			
B_e^i	5015.37(16)	1328.8427(47)	1061.4123(38)			

Table S13 – Computed values of ΔB_{vib}^{i} and B_e^i for 2-MI…H_2O.

See text for definitions of B_0^i, ΔB_{vib}^{i} and B_e^i. Values of B_0^i rotational constants are as shown in Table S8 (*a*, *b* and *c* denote the respective inertial axes). Values of ΔB_{vib}^{i} are computed by density functional theory using the B3LYP functional, the partially-augmented double-ζ jun-cc-pVDZ basis set and Grimme's DFT-D3 scheme with the Becke-Johnson (BJ) damping function for the treatment of dispersion effects. Values of B_e^i are determined by subtracting ΔB_{vib}^{i} from B_0^i according to the equation shown (Eqn 3 in the text).

$$B_e^i = B_0^i - \Delta B_{vib}^{i} = B_0^i + \frac{1}{2}\sum_r \alpha_r^i$$

	2-MI…$H_2{}^{16}O$			2-MI…$H_2{}^{18}O$		
	a	*b*	*c*	*a*	*b*	*c*
B_0^i	4233.93(21)	1732.5621(61)	1240.3104(66)	4216.088(93)	1628.8335(43)	1184.7799(35)
$\sum_r \alpha_r^i$	157.024	31.30892	30.74646	160.0268	30.22398	29.87129
$-\Delta B_{vib}^{i}$	78.512	15.65446	15.37323	80.0134	15.11199	14.93565
B_e^i	4312.44(21)	1748.2166(61)	1255.6836(66)	4296.101(93)	1643.9455(43)	1199.7155(35)
	2-MI…DOH			2-MI…HOD		
	a	*b*	*c*	*a*	*b*	*c*
B_0^i	4212.776(60)	1707.6301(27)	1225.7031(25)	4184.178(58)	1661.6617(20)	1200.1864(24)
$\sum_r \alpha_r^i$	131.60096	27.55827	27.13067	160.6056	29.0206	29.72541
$-\Delta B_{vib}^{i}$	65.80048	13.77914	13.56534	80.3028	14.5103	14.86271
B_e^i	4278.576(60)	1721.4092(27)	1239.2684(25)	4264.481(58)	1676.1720(20)	1215.0491(24)
	2-MI…D_2O					
	a	*b*	*c*			
B_0^i	4165.477(74)	1639.0453(25)	1186.8983(28)			
$\sum_r \alpha_r^i$	133.82315	25.01936	25.77635			
$-\Delta B_{vib}^{i}$	66.9116	12.50968	12.88818			
B_e^i	4232.389(74)	1651.5550(25)	1199.7865(28)			
